# Does high gonial angle increases the risk of mandibular angle fracture? A digital orthopantomographic study 

**DOI:** 10.4317/jced.60003

**Published:** 2022-12-01

**Authors:** Alah-Dawood Al-Dawoody

**Affiliations:** 1 BDS, MSc, PhD, MFDSRCPS(Glasg.), MFDRCSI. Assistant professor of Orthodontics, Department of Dentistry. Al-Manara College for Medical Sciences. Mysan, Republic of Iraq

## Abstract

**Background:**

Fracture of mandibular angle comprises up to one third of mandibular fractures. Many local anatomical factors may increase the risk of such fractures. This study was conducted to investigate the risk of angle fractures with gonial angle measurements.

**Material and Methods:**

A retrospective cross-sectional study was conducted on digital panoramic radiographs of 120 patients with isolated mandibular fractures. The patients were categorized into mandibular angle and other nonangle fracture groups. The gonial angle, antegoneal angle, ramus height was measured using Image J software.

**Results:**

Out of 120 isolated mandibular fractures, 40 (33.33%) are angle fractures and 80 (66 .67%) are other nonangle isolated fractures. Seventy- five percent of angle fractures and 85% of non-angle fractures occurred in males. The average age of angle fracture patients was 28.34±5.2 years and 27.37 ±4.9 years for the non-angle fractures with no significant difference (*p*=0.31). The gonial angle of the angle fractures was 127.60 º ±4.93 º which is 6 degrees greater than the non-angle fractures. The antegonial angle of the angle fractures was 160.67±5.38º which is 5.2 º less than the non-angle fractures. The ramus height in the angle fractures was 18.97±3.7 mm which is 2.5 mm shorter than the nonangle fractures. The difference in these three measurements was highly significant (*p*<0.0001). About 45% of angle fractures and 19% of non-angle fractures occurred in the high gonial angle.

**Conclusions:**

The presence of high mandibular angle doubles the risk of mandibular angle fracture.

** Key words:**Angle fracture, gonial angle, Image J, mandiblular fractures, orthopantomograph.

## Introduction

Mandibular fractures are one of the most common fractures, occur more frequently in men, and are often associated with other injuries such as cervical spine fractures and neurological injuries (1). Mandibular fractures are the second most common facial fractures after nasal fractures (2) and account for more than half of all maxillofacial fractures (3). The mandible is relatively prominent with respect to the skull and is prone to fractures. Therefore, mandibular fractures, especially in the condyle region, act as a defense mechanism and prevent serious trauma to upper sensitive areas such as the brain and skull (4).

Mandibular angle fractures comprise 30% of all mandibular fractures (5). The mandibular angle forms where the mandibular body joins the ramus and the anterior margin of masseter muscle forms its anterior limit (6). Many anatomical and mechanical factors may increase the risk of angle fracture, such as abrupt change in the direction from horizontal body to vertical rami, impacted third molars, reduced bone volume and the direction of pterygomassetric sling pull at the angle region (7).

Three different terms have been used to describe the mandibular angle region. The clinical angle is where the alveolar process joins the mandibular ramus at the beginning of internal oblique ridge. The surgical angle is the region where the mandibular body abuts the ramus at the origin of external oblique ridge. The gonial angle indicates the point of intersection of the lower border of mandibular body with the posterior border of the ramus. The mandibular angle fracture is defined as a fracture, situated distal to the second molar, which extends downward from any point on the curve formed by the junction of the body and ramus, in the retromolar region, to any point on the curve where the inferior border of the body and posterior border of the ramus unite (8). There are many anatomical and biomechanical factors that are associated with mandibular angle fractures such as the severity and direction of the impact forces; bone density, mass, and irregular anatomic structure, and wide gonial angle (9).

The gonial angle is considered as an important anthropometric variable that reflects the vertical growth pattern (10) and also determines the direction of rotation of the mandible (11). In addition, gonial angle is valuable measurement in deciding for premolar extraction during treatment of Class II malocclusion (12), and the decision to execute orthognathic surgery on skeletal Class III malocclusion (13). Gonial angle is also useful for age estimation in the identification of skeletal remains (14).

The gonial angle can be measured directly on the face, on radiographs, or photographs. According to the divergence of gonial angle, the skeletal pattern can be classified as a high or low angle. Increased divergence of gonial angle and lower anterior facial height occurs when the mandible grows in downward and backward direction. Contrary to this, upward and forward growth of the mandible will lead to convergent gonial angle and decreased lower anterior facial height (11). The gonial angle also influence the bony architecture of the mandibular angle region (15). Individuals with high gonial angle have a relatively low occlusal forces, which in turn lead to reduced cortical bone thickness, rendering it more vulnerable to fractures (16). The presence of an impacted mandibular third molar also contributes to further weaken of the angle region (17).

 This study studied the relation between gonial angle and mandibular angle fractures.

## Material and Methods

This a retrospective cross sectional observational study of patients presented to a university affiliated hospital for treatment of isolated mandibular fractures. Patients included in the study were aged 20 or older, and had clinical records and high quality digital panoramic radiographs.

-Exclusion criteria

The exclusion criteria included radiographs with poor contrast, distortion, magnification, positional or exposure errors. In addition, edentulous patients, patients with comminuted fractures, pan facial fractures, dentoalveolar fractures, more than one mandibular fracture, history of orthodontic, orthpaedic or orthognathic surgery were also excluded. Patients with pathological lesions, facial asymmetry, postoperative trauma cases were also excluded.

-Experimental

The data for descriptive analysis was derived from medical records and included the following; age & sex of the patient, cause of trauma, anatomical location of fracture (symphysis, parasymphysis, body, angle, condyle), gonial angle size, presence or absence of impacted mandibular third molar.

**Figure 1:**
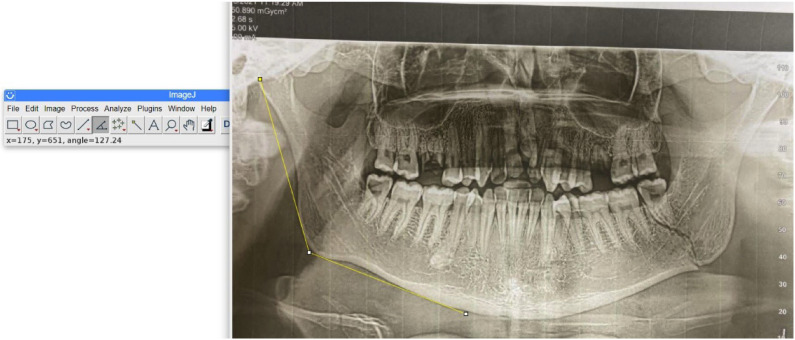
Measuring the gonial angle on digital panoramic radiograph using Image J program.

A diagnosis of a mandibular angle fracture was made when the fracture line was present posterior to the second molar, passing through the mandibular body to the lower border. Image J software was used to measure the gonial, antegonial angles and ramus length (Fig. 1). The gonial angle was measured on the nonfracture side by drawing a tangent to the posterior border of the ramus (articular-gonion) and tangent to the lower border of the mandible (gonion-gnathion). The gonial angle was categorized as Normal ((121.8 ± 6.2), high (> 128), and low angle (< (115.6).

**Figure 2:**
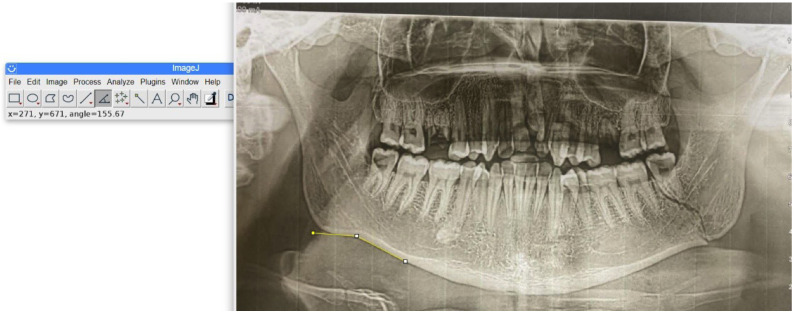
Measuring antegonial angle on digital panoramic radiograph using Image J program.

The antegonial angle was measured by tracing two lines parallel to the antegonial region that intersected at the deepest point of the antegonial notch and the ramus height was estimated by measuring the distance between Articulare and Gonion (Fig. 2).

-Statistical analysis

The data were analyzed using statistical software (S.P.S.S. Version 21). Fischer exact test was used to assess the effect of age and gender on angle and nonangle fractures. Unpaired T test was used for comparison of gonial and antegonial angles and ramus height of angle and non-angles fractures. The correlation between angle fractures and gonial angle value was assessed by Cramer’s correlation V test. Statistical significance was set at a *P* value ≤ 0.05.

## Results

The age and sex distribution are shown in  Table 1. Out of 120 isolated mandibular fractures, 40 (33.33%) are angle fractures and 80 (66 .67%) are other nonangle isolated fractures. Seventy- five percent of angle fractures and 85% of non-angle fractures occurred in males. The sex difference is highly significant (*p*<0.05). The average age of angle fracture patients was 28.34±5.2 years and 27.37 ±4.9 years for the non-angle fractures with no significant difference being noted (*p*=0.31). Although about 45% of both angle and non-angle fractures occurred in the age group of 20-29 years, no significant difference was present among the four age groups (*p*=0.81).

**Table 1:**
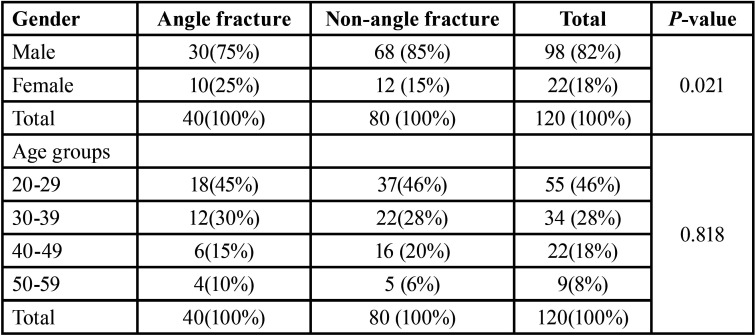
Age and sex distribution of angle and non-angle fractures.

Gonial angle and antegonial angle values and ramus height measurements in the angle and non-angle fractures are shown in Table 2. The gonial angle of the angle fractures patients was 127.60±4.93 which is 6 degrees greater than the other non-angle isolated mandible fractures. The antegonial angle of the angle fractured patients was 160.67±5.38 which was 5.2 less than the non-angle fractures cases. The ramus height (Ar-Go) in the angle fractures patients was 18.97±3.7 mm which was 2.5 mm shorter than the other non-angled fractured cases. The difference in the gonial and antegonial angles and ramus height between the angle and non-angle fractured mandibles was highly significant (*p*<0.0001). Correlation of angle and other non-angle fractured mandibles with gonial angle is shown in Table 3. About 45% of angle fractures occurred in the high gonial angle patients and 47% of non-angle fractures occurred in the low gonial angle patients. The difference between the two groups was significant (*p*=0.000). Cramer’s V correlation of angle and other non-angle fractures with gonial angle measurement was medium (V=0.35).

**Table 2:**
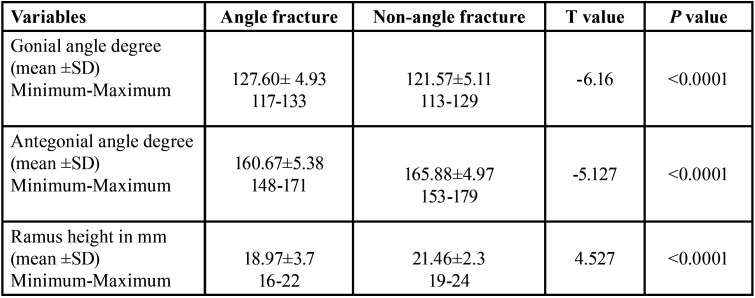
Gonial and antegonial angles and ramus height in the angle and nonangle fractures.

**Table 3:**
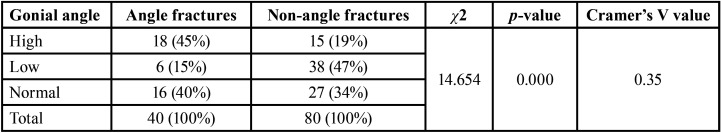
Correlation gonial angle with angle fracture risk.

## Discussion

Mandibular angle fracture has the highest postsurgical complication and among all types of mandibular fracture (18). Angle fractures are the second most common mandibular fractures, after condylar fractures (19). The angle region is the first most frequent site for fractures caused by contact sports accidents, the second most common fractures caused by inter personal violence and the third most fractured region in road traffic accidents (20).

Mandibular angle fracture usually commences at the upper border of the mandible, where the anterior border of the ramus meets the mandibular body, usually in the third molar region, and extends downward to the inferior border or backward, in the gonial angle region (8). Mandibular angle fractures may be favourable or unfavuorable, depending on the direction of fracture line, in the horizontal and vertical planes, and the degree of displacement of the proximal and distal fractured segments (21).

The susceptibility of mandibular angle to fractures could be attributed to the anatomic weakness of this region, due to its abrupt curvature and changes in the direction of osseous trabeculae. Factors such as impacted third molars, reduced mandibular height, and poor bone quality could also reduce the strength of mandible, making it more susceptible to fracture (22).

 Gonial angle is an important angular measurement, used to assess the growth trend and pattern (23). Based on the degree of gonial angle, the mandible can be considered as having low or high angle (24). A high gonial angle indicates clockwise rotations of the mandible, whilst low gonial angle designates anticlockwise growth rotation of the mandible (25).

Radiographic examination employing cephalometric or panoramic radiographs is used in the clinical context to determine the gonial angle. The orthopantomograph is used in this investigation to locate the fracture site and measure the gonial, antegonial, and ramus heights. According to Abdul Rehman *et al*. (26), a panoramic radiograph allows for more precise measuring of the gonial area without the influence of superimposed structures seen in lateral cephalograms. Many investigations have found that the right and left gonial angles are essentially identical, with any deviations considered minor (27). As a result, the nonfractured side’s gonial angle was taken into account in this investigation. The gonial angle has been found to be the most accurate measurement obtained from an orthopantomograph. This is most likely due to the fact that it is an angular rather than a linear measurement, and hence is unaffected by magnification (28).

The results of this study showed that the gonial angle of patients with unilateral angle fractures is 127.60 which is 6 degrees larger than the gonial angle of other mandibular fracture sites. In addition, 45% of angle fractures occurred in high angle cases and 47% of non-angle fractures occurred in the low angle cases The Cramer’s V correlation showed a medium effect of high gonial angle on the occurrence of mandibular angle fractures.

The findings of this study are consistent with the results of other studies. Panneerselvam *et al*. (2), found that the gonial angle was 4.5° greater than other nonangle fractures. Dhara *et al*. (29), demonstrated that the gonial angle was 10.2° larger in the angle fractures as compared to non-angle fractures. Elias *et al*. (30), in a CT-based study, detected that the gonial angle of mandibular angle fractures was 13.2 ° greater than nonangle fractures. Semel *et al*. (31), discovered that angle fractures had a larger gonial angle than other fracture locations. Elavenil *et al*. (32), also concluded that the gonial angle was 4.5° greater in angle fractures.

The higher vulnerability to angle fractures in high-angle patients was previously attributed to lower bite forces, which result in less cortical bone thickness at the angle region. Furthermore, it has been demonstrated that in high angle cases, the height of the mandible at the ramus and angle area is much lower than in normal persons (33). The current study found that ramus height and antegonial angle are considerably lower in high angle fracture patients than in normal and low angle cases.

## Conclusions

The gonial angle has a significant impact on mandibular angle fractures. Angle fractures are associated with a high gonial angle, whereas non-angle fractures are associated with a low gonial angle. Angle fractures have a smaller ramus height and antegonial angle than non-angle fractures.
